# Combination of computed tomography imaging pattern and severity of respiratory failure as factors associated with prognosis for acute exacerbation of idiopathic chronic fibrosing interstitial pneumonia

**DOI:** 10.1371/journal.pone.0279878

**Published:** 2023-02-24

**Authors:** Keito Enokida, Takahisa Takihara, Yukihiro Horio, Noriko Nakamura, Naotaka Kutsuzawa, Mari Takahashi, Fuminari Takahashi, Sakurako Tajiri, Yoko Ito, Koichiro Asano

**Affiliations:** 1 Division of Pulmonary Medicine, Department of Medicine, Tokai University School of Medicine, Isehara, Japan; 2 Division of Pulmonary Medicine, Department of Medicine, Tokai University Oiso Hospital, Oiso, Japan; 3 Department of Radiology, Tokai University School of Medicine, Isehara, Kanagawa, Japan; Stanford University School of Medicine, UNITED STATES

## Abstract

**Background and objectives:**

The prognosis of idiopathic chronic fibrotic interstitial pneumonitis (CFIP) in patients with acute exacerbation (AE) is variable. We examined whether the imaging pattern on thoracic computed tomography (CT) or the severity of respiratory failure with AE-CFIP is associated with short-term prognosis.

**Methods:**

Patients admitted to two university hospitals were retrospectively analyzed and divided into derivation and validation cohorts. The distribution of newly appearing parenchymal abnormalities on thoracic CT was classified into peripheral, multifocal, and diffuse patterns. Respiratory failure was defined as severe if a fraction of inspired oxygen ≥ 0.5 was required to maintain percutaneous oxygen saturation ≥ 90% on admission. Factors associated with 90 day-mortality were analyzed using univariate and Cox proportional hazard regression.

**Results:**

In 59 patients with AE-CFIP of the derivation cohort, diffuse pattern on CT was associated with higher mortality within 90 days (43%) than peripheral/multifocal pattern (17%, p = 0.03). Additionally, compared with non-severe failure, severe respiratory failure was associated with higher mortality (47% vs. 21%, p = 0.06). Cox proportional hazard regression analysis demonstrated that a combination of diffuse pattern on CT and severe respiratory failure was associated with the poorest prognosis (hazard ratio [HR] 3.51 [interquartile range 1.26–9.80], p = 0.016) in the derivation cohort, which was confirmed in the validation cohort (n = 31, HR 4.30 [interquartile range 1.51–12.2], p = 0.006).

**Conclusion:**

The combination of imaging pattern on thoracic CT and severity of respiratory failure was associated with the prognosis of idiopathic AE-CFIP.

## Introduction

Acute exacerbation (AE) accompanied by new ground-glass opacity and consolidation in the lungs along with rapid acceleration of respiratory failure are the major causes of mortality in patients with idiopathic pulmonary fibrosis (IPF). Approximately half of the deaths of IPF patients are associated with AE, and the estimated median survival time of IPF patients who have developed AE is 3–4 months [1–5]. AE also occurs in patients with idiopathic chronic fibrotic interstitial pneumonitis (CFIP) other than IPF [6, 7].

Several studies have shown that thoracic computed tomography (CT) imaging patterns and distribution are useful for predicting the prognosis of AE in patients with IPF. Akira et al. [8] defined three distribution patterns of ground-glass opacity or consolidation newly developed during AE of IPF: peripheral, multifocal, and diffuse. They examined 58 patients with AE-CFIP and demonstrated that cases with diffuse pattern of ground-glass opacity or consolidation exhibit a significantly worse prognosis compared to those with a peripheral/multifocal pattern [8]. Another scoring system for CT imaging was proposed by Ichikado et al. [9], who semi-quantitatively evaluated the extent of ground-glass opacity, consolidation, bronchiectasis, and honeycomb. An Ichikado score of ≥ 245 has been attributed with a significantly poor prognosis in patients with AE of IPF [10].

In contrast, several other studies have shown that oxygenation status is a prognostic factor for AE in patients with IPF. Parameters for oxygenation during AE, particularly partial pressure of oxygen in arterial blood (PaO_2_)/ fraction of inspired oxygen (FiO_2_) < 200, have been reported to be associated with poor prognosis [2, 5, 6, 10–19]. The PaO_2_/FiO_2_ ratio is also a well-established parameter for classification of the severity of acute respiratory distress syndrome (ARDS) [20], which presents with the same pathological changes in the lungs as AE-IPF. ARDS with a PaO_2_/FiO_2_ ratio < 100 is considered severe and is associated with a poor prognosis regardless of the treatment [20].

Though several biomarkers, including concentration of lactate dehydrogenase, C-reactive protein, Krebs von Lungen-6 (KL-6) in serum, and neutrophil and lymphocyte counts in bronchoalveolar lavage fluid (BALF) have also been linked to poor prognosis of AE-IPF [2, 5, 11, 13, 15, 17, 21–23], the results are inconsistent. In the present study, therefore, we focused on the two parameters, imaging pattern on thoracic CT and oxygenation status, to confirm whether either of them or a combination of the two could provide information toward predicting short-term prognosis of patients with CFIP following AE.

## Materials and methods

### Subjects

Patients who were admitted to Tokai University Hospital, a tertiary university hospital in Japan, due to AE-CFIP between April 2006 and March 2018, were retrospectively analyzed as a derivation cohort. Patients who were admitted to Tokai University Oiso Hospital during the same period and those who were admitted to Tokai University Hospital due to AE-CFIP between April 2018 and August 2020 were retrospectively analyzed as a validation cohort.

Idiopathic CFIP, including IPF, unclassifiable interstitial pneumonia, and non-specific interstitial pneumonia, was diagnosed based on multidisciplinary discussion (MDD) with multiple pulmonologists and a radiologist to assess patients’ clinical history, physical examination, laboratory data, and radiographic findings on high-resolution CT (HRCT). Patients with secondary conditions due to connective tissue disease, hypersensitivity pneumonitis, and sarcoidosis were excluded; those with neoplasms undergoing active treatments, including cytotoxic chemotherapy, molecular-targeted drugs, or immune checkpoint inhibitors were also excluded.

AE-CFIP was diagnosed based on modified AE-IPF criteria [24], which was as follows: 1) previous or concurrent diagnosis of CFIP, 2) unexplained worsening of dyspnea within one month of diagnosis; 3) new bilateral ground-glass opacity and/or consolidation on chest radiograph or CT; and 4) deterioration not fully explained by cardiac failure or fluid overload.

This study was reviewed and approved by the Institutional Review Board of Tokai University Hospital (17R-198/18R-241/20R-230) and Tokai University Oiso Hospital (18R-241), implemented in compliance with the Declaration of Helsinki. Informed consent was obtained in the form of opt-out on the website. No patient declined to participate in the study.

### Clinical and laboratory parameters

Data associated with clinical and laboratory parameters within 12 months prior to and after the onset of AE-CFIP were collected from medical charts. The systemic inflammatory response syndrome (SIRS) score at the time of admission for AE-CFIP was determined based on body temperature, heart rate, respiratory rate, and white blood cell counts, as previously reported [25]. While the levels of serum KL-6 were measured using an electrochemiluminescence immunoassay and a Lumipulse G1200 Analyzer (Rebio, Fuji, Japan), commercially available enzyme-linked immunosorbent assay kits (RayBiotech, Norcross, GA, USA) were used to evaluate the levels of surfactant protein D (SP-D). Spirometry data obtained using a Super Spiro DISCOM-21FX III spirometer (CHEST Corp., Tokyo, Japan) by well-trained clinical technicians within one year prior to AE-CFIP were used in the analysis. The predicted values of forced vital capacity (FVC) and forced expiratory volume in one second (FEV_1_) were calculated using equations proposed by the Japanese Respiratory Society [26].

### Severity of respiratory failure classified by oxygenation status

Respiratory failure severity at admission was defined according to the minimum FiO_2_ required to maintain percutaneous oxygen saturation (SpO_2_) ≥ 90% on admission. The FiO_2_ during oxygen therapy was estimated using the conversion formula described below [27].

Respiratory failure was defined as non-severe or severe when the patients required FiO_2_ < 0.5 or ≥ 0.5, respectively, to maintain SpO_2_ ≥ 90%. As per the Berlin definition, the severe group corresponds to moderate or severe oxygenation disorder (PaO_2_/FiO_2_ < 200) [20].

### Radiographic scoring of thoracic CT

Thoracic HRCT with 1.5 mm-thick axial sections was obtained at 1-cm intervals throughout the entire thorax in the inspiratory phase prior to the onset of AE. CT images were classified according to the consensus reading by three pulmonologists blinded to the prognosis of each case as usual interstitial pneumonia (UIP)/probable UIP/indeterminate for UIP/alternative diagnosis based on the 2018 ATS/ERS/JRS/ALAT international diagnostic guidelines [28].

Newly appeared ground-glass attenuation (GGA) and consolidation in the lungs on CT at AE-CFIP were classified into three patterns, namely peripheral, multifocal, and diffuse, according to the definition by Akira et al. [8]. The Ichicado score was determined as follows [9]: CT images of the lungs were evaluated at six parts; left and right lungs above the level of the tracheal carina (the upper zone), below the level of the inferior pulmonary vein (the lower zone), and between the upper and lower zones (the middle zone). Each part of the lung field was scored from 1 to 6 as follows: score 1, normal attenuation (spared area); score 2, GGA without traction bronchiectasis or bronchiolectasis; score 3, consolidation without traction bronchiectasis or bronchiolectasis; score 4, GGA with traction bronchiectasis or bronchiolectasis; score 5, consolidation with traction bronchiectasis or bronchiolectasis; and score 6, honeycombing. The extent of each abnormality was determined by visually estimating the percentage (to the nearest 10%) of the affected lung parenchyma in each zone. The abnormality score for each zone was calculated by multiplying the percentage area by the point value (1–6). For each patient, the six zone scores were averaged to determine the total score for each abnormality, and the overall HRCT score was obtained by adding six average values. Area of newly appeared GGA and consolidation (the percentage of the affected lung parenchyma) was also evaluated at six parts of the lungs as described above.

### Statistical analysis

Data are presented as mean ± standard deviation for continuous variables and as numbers and percentages for categorical data. Group comparisons were made using the Mann-Whitney U test for continuous variables and Fisher’s exact test for categorical variables. Kaplan-Meier survival curves and log-rank tests were employed to evaluate survival. In addition, a log-rank trend test was conducted to confirm the changes in trends related to 90-day mortality. When the severity of respiratory failure and CT pattern were analyzed in combination, patients were categorized into three groups: cases with non-severe respiratory failure and non-diffuse CT pattern, those with severe respiratory failure and diffuse pattern, and the discordant group with either non-severe respiratory failure and diffuse CT pattern or severe respiratory failure and non-diffuse CT pattern. Cox proportional hazard regression analysis for 90-day survival in the derivation cohort was performed using the parameters identified by p < 0.1 in the univariate analyses and no missing data. Hazard ratios (HRs) were presented as median and interquartile range (IQR). Statistically significant parameters in the analysis of the derivation cohort were applied to the validation cohort. Patients with IPF in the derivation and validation cohorts (an IPF cohort) were also used for the sensitivity analysis. All analyses were performed using the IBM SPSS Statistics software ver. 27 (IBM Corp., Armonk, NY, USA). The level of statistical significance was set at p < 0.05.

## Results

### Subjects

We identified 59 patients (median age, 75 years; 53 men) admitted due to AE-CFIP during the study period in the derivation cohort. For the validation cohort, 34 patients with AE-CFIP were found to be eligible in the two institutes. Two patients, one each from in the derivation and validation cohorts were lost to follow-up within 90 days, and were excluded from the univariate analysis of 90-day mortality.

### Clinical characteristics and radiographic findings

**Fig 1:**
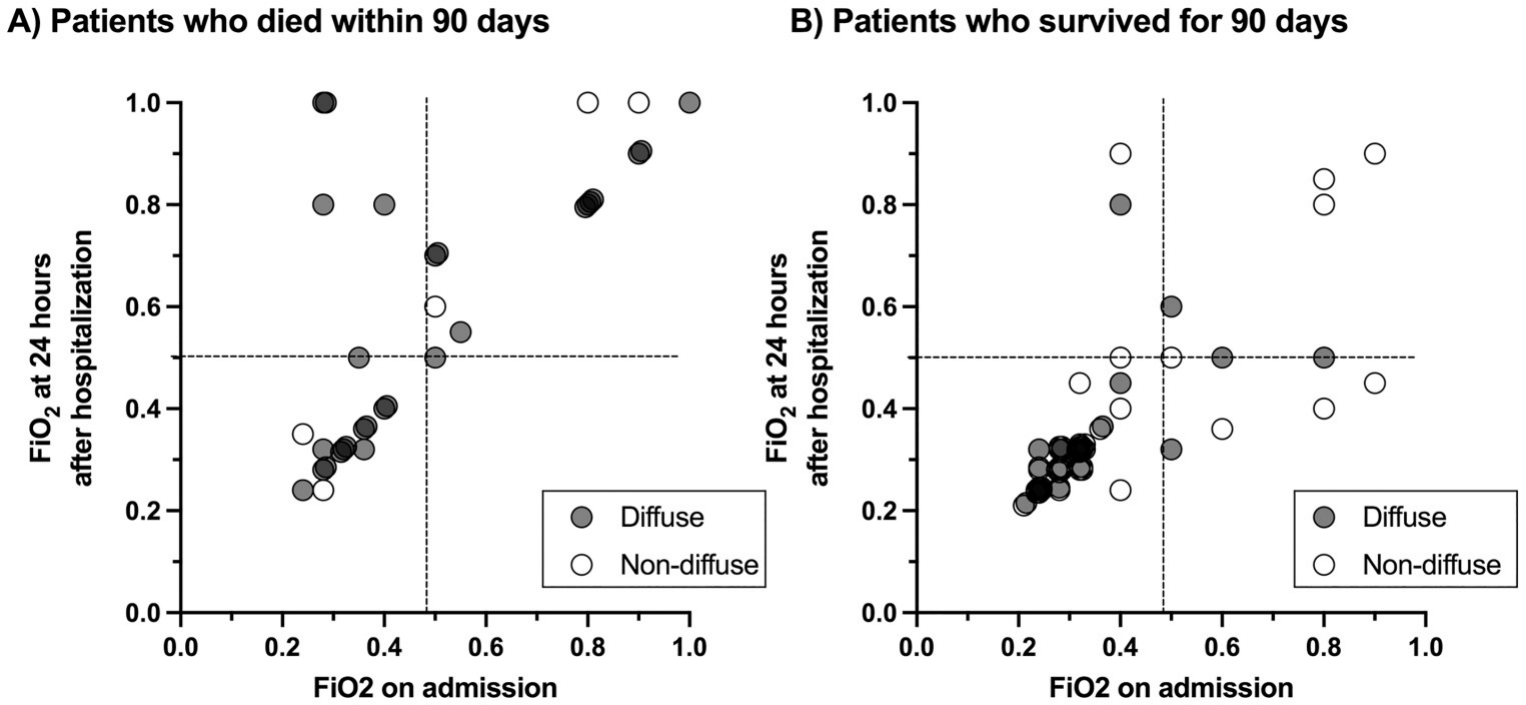
Scatter plot of the fraction of inspired oxygen (FiO_2_) required to maintain percutaneous oxygen saturation (SpO_2_) ≥ 90% on admission and at 24 hours after hospitalization. The data were obtained from the combined dataset of the derivation and validation cohorts; it includes patients who died (n = 33, A) and those who survived (n = 68, B) within 90 days. Patients who presented with a diffuse pattern on CT at admission are demonstrated as closed circles and those presented with peripheral/multifocal patterns are demonstrated as open circles.

According to the classification of the severity defined by FiO_2_ required to maintain SpO_2_ ≥ 90% on admission, there were 19 (32%) and 7 (21%) subjects with severe respiratory failure requiring FiO_2_ ≥ 0.5, in the derivation and validation cohorts, respectively. In 12 patients (13%), the respiratory failure grade changed within 24 hours after hospitalization. Specifically, eight patients worsened and four improved (Fig 1). With regards to the demographic data and clinical characteristics within 12 months before AE (Table 1), though the patients in the validation cohort were older, the lung functions and CT images prior to AE were not significantly different between the groups. The proportion of IPF was also equivalent in the two cohorts (69% vs. 62%).

**Table 1:** Demographic data at baseline and at acute exacerbation in derivation and validation cohort.

	Derivation cohort (n = 59)	Validation cohort (n = 34)	p- value
At baseline			
Age, years	74.0 ± 6.7	78.0 ± 6.8	0.01
Male, n (%)	53 (90%)	25 (74%)	0.04
Smoking status			
Smokers, n (%)	42 (71%)	22 (65%)	0.95
Pack year	42 ± 57	29 ± 26	0.17
Pulmonary function tests			
%FVC	63 ± 22 (n = 26)	74 ± 26 (n = 14)	0.23
KL-6 (U/mL)	1379 ± 869 (n = 46)	1171 ± 1408 (n = 20)	0.02
Treatment			
Long-term oxygen therapy, n (%)	14 (24%)	7 (21%)	0.73
Prednisolone, n (%)	14 (24%)	6 (18%)	0.49
Prednisolone, dose (mg/day)	15 ± 7 (n = 14)	10 ± 5 (n = 6)	0.18
Immunosuppressive therapy, n (%)	3 (5%)	1 (3%)	0.63
Antibiotics, n (%)	11 (19%)	8 (24%)	0.58
Antifibrotic therapy, n (%)	4 (7%)	4 (10%)	0.41
HRCT findings			
Definite/probable UIP	27 (46%)/15 (25%)	8 (24%)/14 (41%)	0.52*
Indeterminate/alternative diagnosis	11 (19%)/6 (10%)	10 (29%)/2 (6%)	
Diagnosis based on MDD			
IPF / Unclassifiable, n (%)	41 (69%)/18 (31%)	21 (62%)/13 (38%)	0.45
On admission due to acute exacerbation			
Respiratory rate (/min)	25 ± 6	23 ± 5	0.14
SIRS score	1.9 ± 1.1	1.4 ± 1.0	0.06
Severe respiratory failure, n (%)	19 (32%)	7 (21%)	0.23
Laboratory data			
White blood cell counts (×10^3^ /mL)	11.1 ± 3.8	10.7 ± 3.6	0.59
C-reactive protein (mg/dL)	8.1 ± 7.1	9.5 ± 7.6	0.39
Lactate dehydrogenase (U/L)	394 ± 157	333 ± 88	0.11
KL-6 (U/mL)	1988 ± 1095	1636 ± 1381	0.01
HRCT findings			
Ichikado score	230 ± 63	174 ± 39	<0.001
Area of GGA and consolidation (%)	67 ± 28	60 ± 38	0.07
CT pattern			
Peripheral/ Multifocal pattern, n (%)	13 (22%) / 17 (29%)	3 (9%) / 5 (15%)	0.017**
Diffuse pattern, n (%)	29 (44%)	26 (76%)	
Treatments			
Drugs			
High-dose prednisolone, n (%)	59 (100%)	34 (100%)	1.00
Glucocorticoid pulse therapy, n (%)	41 (69%)	21 (62%)	0.45
Immunosuppressants, n (%)	4 (7%)	1 (3%)	0.29
Antibiotics, n (%)	39 (66%)	10 (29%)	0.18
Diuretics, n (%)	6 (10%)	4 (10%)	0.81
Oxygen therapy			
Supplemental oxygen, n (%)	59 (100%)	34 (100%)	1.00
High flow nasal cannula, n (%)	9 (15%)	9 (26%)	0.19
IPPV, n (%)	3 (5%)	0 (0%)	0.18

FVC, forced vital capacity; GGA, ground-glass attenuation; HRCT, high-resolution computed tomography; IPPV, intermittent positive pressure ventilation; IPF, idiopathic pulmonary fibrosis; KL-6, Krebs von Lungen-6; MDD, multidisciplinary discussion; SIRS, systemic inflammatory response syndrome; UIP, usual interstitial pneumonia

* UIP/probable UIP vs. indeterminate UIP/alternative diagnosis,

** Peripheral/multifocal pattern vs. diffuse pattern

Some clinical characteristics upon admission due to AE (Table 1) were significantly different between the subjects in the two cohorts. Serum KL-6 levels were lower in the validation cohort. Among radiographic parameters, the Ichikado score was significantly lower in the validation cohort, whereas newly appeared parenchymal abnormalities were more often observed as a diffuse pattern in the validation cohort than in the derivation cohort (76% vs. 44%).

### Treatments

Every patient was treated with high-dose prednisolone with or without corticosteroid pulse therapy or immunosuppressants. All of them required oxygen therapy at the onset of AE, and for 12 patients (20%) in the derivation cohort and 9 (26%) in the validation cohort, high-flow nasal cannula or invasive positive-pressure ventilation was used for the treatment of respiratory failure. There were no significant differences in pharmacotherapy or oxygen therapy between the groups (Table 1).

### Derivation cohort 90-day mortality

Seventeen patients (29%) died within 90 days after AE (non-survivors), including 8 patients (21%) with non-severe respiratory failure and 9 patients (47%) with severe respiratory failure (p = 0.06, Table 2). According to the CT pattern, three patients had a peripheral pattern (18%), two had multifocal patterns (12%), and 12 with diffuse patterns (71%) died within 90 days, suggesting the poorest prognosis in the cases with diffuse pattern (p = 0.03). Other factor associated with poor prognosis in univariate analysis were UIP or probable UIP findings (p = 0.01) and prior immunosuppressive treatments (p = 0.02).

**Table 2:** Comparison of survivors and non-survivors in derivation cohort.

	Survivors (n = 41)	Non-survivors (n = 17)	p- value
At baseline			
Age, years	75 ± 7	74 ± 6	0.76
Male/female, n (%)	37 (90%)	15 (88%)	0.82
Smoking status			
smoker, n (%)	32 (78%)	9 (53%)	0.05
Pulmonary function tests			
%FVC	69 ± 19 (n = 19)	46 ± 24 (n = 8)	0.04
KL-6 (U/ml)	1333 ± 842 (n = 30)	1466 ± 940 (n = 16)	0.75
HRCT findings, n (%)			
Definite/Probable UIP	17 (41%) / 8 (20%)	10 (59%) / 6 (35%)	0.01*
Indeterminate/alternative diagnosis	10 (24%) / 6 (15%)	1 (6%) / 0 (0%)	
Diagnosis based on MDD			
IPF/Unclassifiable, n (%)	25 (61%) / 16 (39%)	15 (88%) / 2 (12%)	0.04
Treatment			
LTOT, n (%)	8 (20%)	5 (30%)	0.42
Prednisolone, n (%)	7 (17%)	6 (38%)	0.13
Prednisolone, dose (mg/day)	12 ± 7 (n = 7)	19 ± 7 (n = 6)	0.01
Immunosuppressive therapy, n (%)	0 (0%)	3 (18%)	0.02
Antibiotics, n (%)	7 (17%)	4 (24%)	0.57
Antifibrotic therapy, n (%)	3 (7%)	1 (6%)	0.85
On admission due to acute exacerbation			
Respiratory rate (/min)	25 ± 6	27 ± 6	0.16
SIRS score	1.8 ± 1.0	2.3 ± 1.1	0.08
Laboratory data			
White blood cell counts (×10^3^ /mL)	10.7 ± 3.7	12.1 ± 4.2	0.17
C-reactive protein (mg/dL)	7.7 ± 7.5	9.4 ± 6.3	0.21
Lactate dehydrogenase (U/L)	395 ± 176	395 ± 106	0.34
KL-6 (U/mL)	1987 ± 937	2005 ± 1465	0.57
Severe respiratory failure	10 (26%)	9 (53%)	0.06
HRCT findings			
Ichikado score	227 ± 58	240 ± 74	0.66
Area of GGA and consolidation (%)	69 ± 28	63 ± 30	0.36
CT pattern			
Peripheral/Multifocal pattern, n (%)	11 (27%) / 14 (34%)	3 (18%) / 2 (12%)	0.03**
Diffuse pattern, n (%)	16 (39%)	12 (71%)	
Treatment			
Drugs			
Glucocorticoid pulse therapy, n (%)	26 (63%)	14 (82%)	0.16
Immunosuppressive therapy, n (%)	3 (7%)	1 (6%)	0.55
Antibiotics, n (%)	25 (61%)	14 (82%)	0.12
Diuretics, n (%)	4 (10%)	2 (12%)	0.82
Oxygen therapy			
High flow nasal cannula, n (%)	6 (13%)	3 (18%)	0.78
IPPV, n (%)	2 (5%)	1 (6%)	0.88

FVC, forced vital capacity; GGA, ground-glass attenuation; HRCT, high-resolution computed tomography; IPPV, intermittent positive pressure ventilation; IPF, idiopathic pulmonary fibrosis; LTOT, long-term oxygen therapy; KL-6, Krebs von Lungen-6; MDD, multidisciplinary discussion; SIRS, systemic inflammatory response syndrome; UIP, usual interstitial pneumonia

* UIP/probable UIP vs. indeterminate UIP/alternative diagnosis,

** Peripheral/multifocal pattern vs. diffuse pattern

**Fig 2:**
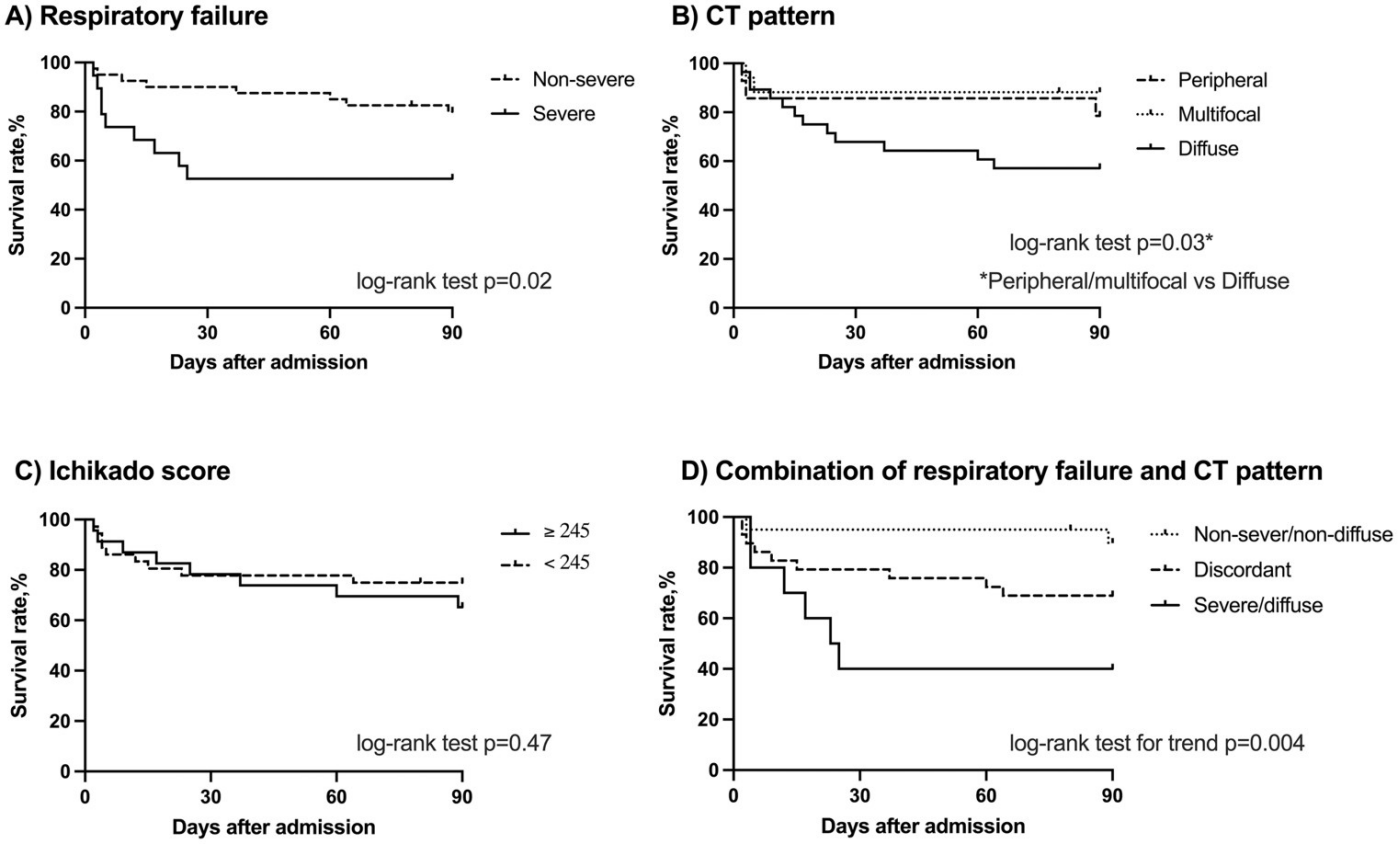
Kaplan-Meier survival curves for patients with acute exacerbation of chronic fibrosing interstitial pneumonia in the derivation cohort. (A) Comparison between the patients with non-severe (dotted line, n = 40) and severe (solid line, n = 19) respiratory failure at hospitalization. (B) Comparison by distribution pattern of newly-appeared opacities on computed tomography (CT); peripheral pattern (dashed line, n = 14), multifocal pattern (dotted line, n = 17), and diffuse pattern (solid line, n = 28). (C) Comparison between the patients with Ichikado score ≥ 245 (solid line, n = 23) and < 245 (dotted line, n = 36). (D) Comparison by the combination of severity of respiratory failure and CT pattern; non-severe respiratory failure and non-diffuse pattern (dotted line, n = 20), severe respiratory failure and diffuse pattern (solid line, n = 10), and discordant group (dashed line, n = 29).

Fig 2 shows the survival rate up to 90 days after AE in the Kaplan-Meier curves. Severe respiratory failure was associated with shorter survival with a median of 5 days (p = 0.02, vs. non-severe respiratory failure, Fig 2A), as was the diffuse pattern on CT with a median survival time of 16 days (p = 0.03, vs. peripheral/multifocal pattern, Fig 2B). On the other hand, there was no significant difference between the subjects with an Ichikado score of 245 or more and < 245 (Fig 2C). When the severity of respiratory failure and CT pattern were analyzed in combination, 2/20 cases (10%) with non-severe respiratory failure and non-diffuse CT pattern died within 90 days, whereas 9/29 cases (31%) in the discordant group and 6/10 cases (60%) with severe respiratory failure and diffuse pattern died within the same period. Survival curve analysis also demonstrated the poorest prognosis in cases with severe respiratory failure and diffuse pattern on CT (p = 0.004, Fig 2D).

### Validation cohort 90-day mortality

**Fig 3:**
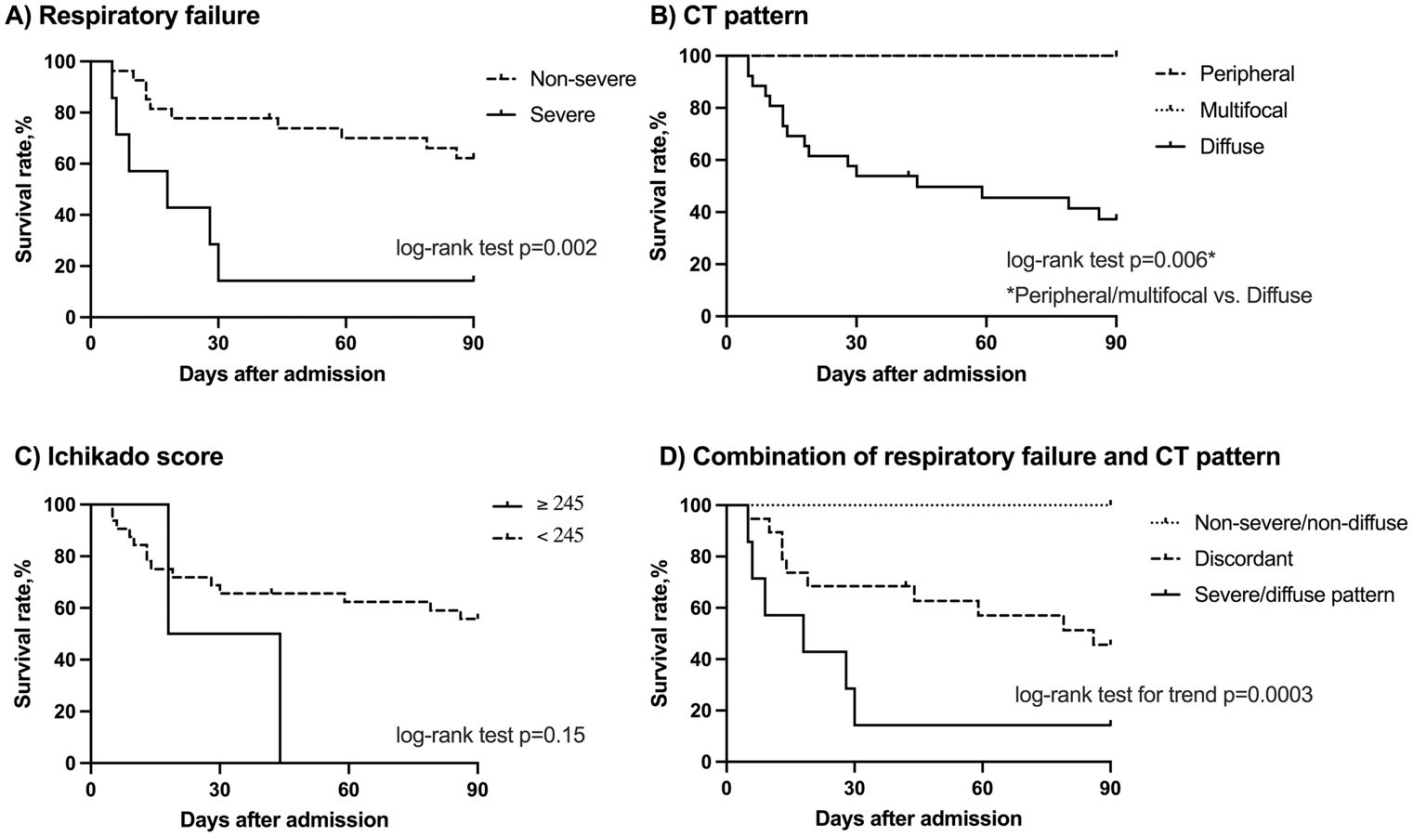
Kaplan-Meier survival curves for patients with acute exacerbation of idiopathic chronic fibrosing interstitial pneumonia in the validation cohort. (A) Comparison between the patients with non-severe (dotted line, n = 27) and severe (solid line, n = 7) respiratory failure on hospitalization. (B) Comparison by distribution pattern of newly-appeared opacities on computed tomography (CT); peripheral pattern (dashed line, n = 3), multifocal pattern (dotted line, n = 5), and diffuse pattern (solid line, n = 26). (C) Comparison between the patients with Ichikado score ≥ 245 (solid line, n = 2) and < 245 (dotted line, n = 32). (D) Comparison by the combination of severity of respiratory failure and CT pattern in the derivation cohort; non-severe respiratory failure and non-diffuse pattern (dotted line, n = 8), severe respiratory failure and diffuse pattern (solid line, n = 7), and discordant group (dashed line, n = 19).

In the validation cohort, 16 patients (48%) died within 90 days after AE. The association demonstrated in the derivation cohort between 90-day mortality and severe respiratory failure or diffuse pattern on CT was replicated in the validation cohort (p = 0.05 and p = 0.02, Table 3). On the other hand, either UIP/probable UIP pattern on CT or prior immunosuppressive treatments were not associated with mortality in the validation cohort (p = 0.44 and 0.79, respectively). Survival curve analysis displayed the poorest prognosis in cases of severe respiratory failure and diffuse pattern on CT (p < 0.001, Fig 3).

**Table 3:** Comparison of survivors and non-survivors in the validation cohort.

	Survivors (n = 17)	Non-survivors (n = 16)	p- value
At baseline			
Age, years	77 ± 7	80 ± 6	0.31
Male, n (%)	13 (76%)	11 (69%)	0.71
Smoking status			
smoker, n (%)	8 (47%)	5 (31%)	0.63
Pulmonary function tests			
% FVC	84 ± 15 (n = 8)	61 ± 32 (n = 6)	0.23
KL-6 (U/ml)	1258 ± 1636 (n = 15)	972 ± 718 (n = 6)	0.84
HRCT findings, n (%)			
Definite/Probable UIP	5 (29%)/5 (29%)	3 (19%)/9 (56%)	0.44*
Indeterminate/alternative diagnosis	6 (36%)/1 (6%)	4 (25%)/0	
Diagnosis based on MDD			
IPF/Others, n (%)	9 (53%)/8 (47%)	12 (75%)/4 (25%)	0.29
Treatment			
LTOT, n (%)	4 (24%)	2 (13%)	0.61
Prednisolone, n (%)	4 (24%)	2 (13%)	0.61
Prednisolone dose (mg/day)	9 ± 6 (n = 4)	11 ± 2 (n = 2)	0.63
Immunosuppressive therapy, n (%)	1 (6%)	0	0.79
Antibiotics, n (%)	3 (18%)	5 (31%)	0.51
Antifibrotic therapy, n (%)	3 (18%)	1 (6%)	0.58
On admission due to acute exacerbation			
Respiratory rate (/min)	22 ± 4	25 ± 5	0.09
SIRS score	1.2 ± 0.9	1.7 ± 1.0	0.18
Laboratory data			
White blood cell counts (×10^3^ /uL)	10.4 ± 2.8	10.6 ± 4.2	0.79
C-reactive protein (mg/dL)	5.5 ± 4.2	13.2 ± 8.5	0.01
Lactate dehydrogenase (U/L)	332 ± 59	338 ± 114	0.71
KL-6 (U/mL)	1618 ± 1196	1707 ± 1645 (n = 14)	0.70
Severe respiratory failure	1 (6%)	5 (31%)	0.05
HRCT findings			
Ichikado score	165 ± 32	185 ± 45	0.44
Area of GGA and consolidation (%)	55 ± 43	68 ± 32	0.11
CT pattern			
Peripheral/ Multifocal pattern, n (%)	3 (18%)/5 (29%)	0 /0	0.02**
Diffuse pattern, n (%)	9 (53%)	16 (100%)	
Treatment			
Drugs			
Glucocorticoid pulse therapy, n (%)	11 (65%)	9 (56%)	0.68
Immunosuppressive therapy, n (%)	0	1 (6%)	0.76
Antibiotics, n (%)	12 (71%)	15 (88%)	0.40
Diuretics, n (%)	1 (6%)	3 (19%)	0.33
Oxygen therapy			
High flow nasal cannula, n (%)	0	8 (50%)	0.01
IPPV, n (%)	0	0	1.00

FVC, forced vital capacity; GGA, ground-glass attenuation; HRCT, high-resolution computed tomography; IPPV, intermittent positive pressure ventilation; IPF, idiopathic pulmonary fibrosis; LTOT, long-term oxygen therapy; KL-6, Krebs von Lungen-6; MDD, multidisciplinary discussion; SIRS, systemic inflammatory response syndrome; UIP, usual interstitial pneumonia

* UIP/probable UIP vs. indeterminate UIP/alternative diagnosis,

** Peripheral/multifocal pattern vs. diffuse pattern

### Prediction of 90-day mortality/survival

We calculated the positive and negative predictive values (PPV/NPV) of the respiratory failure grade and the combination of the respiratory failure grade and CT pattern, to predict 90-day mortality/survival using the combined dataset of derivation and validation cohorts (n = 91, Table 4). The PPV of severe respiratory failure on admission for predicting 90-day mortality was 56%, which increased to 79% when combined with a diffuse pattern on CT. Re-evaluation of respiratory failure 24 hours after hospitalization also increased PPV (66%), but not as much as the evaluation combined with CT pattern. Non-severe respiratory failure on admission or at 24 hour after hospitalization could predict 90-day survival with PPVs of 72% and 78%, respectively, whereas PPV increased to 92% when non-severe respiratory failure was combined with a non-diffuse pattern on CT. Even in the presence of severe respiratory failure on admission, 90-day survival rate increased from 44% to 70% in the absence of a diffuse pattern on CT.

**Table 4:** Positive and negative predictive values to predict 90-day mortality or survival.

	Prediction of 90-day mortality		Prediction of 90-day survival	
	PPV	NPV	PPV	NPV
Respiratory failure evaluated on admission				
Severe respiratory failure alone	56%	72%	44%	28%
Combination of severe respiratory failure and diffuse pattern on CT	79%	72%		
Combination of severe respiratory failure and non-diffuse pattern on CT			70%	64%
Non-severe respiratory failure alone			72%	56%
Combination of non-severe respiratory failure and non-diffuse pattern on CT			92%	48%
Respiratory failure evaluated at 24 hours after hospitalization				
Severe respiratory failure alone	66%	78%	34%	22%
Combination of severe respiratory failure and diffuse pattern on CT	76%	76%		
Combination of severe respiratory failure and non-diffuse pattern on CT			63%	65%
Non-severe respiratory failure alone			78%	66%
Combination of non-severe respiratory failure and non-diffuse pattern on CT			93%	48%

CT, Computed tomography; PPV, Positive predictive value; NPV, Negative predictive value

### Cox proportional hazard regression analysis

Cox proportional hazard regression analysis adjusted for age and sex was performed using the parameters identified by p < 0.1 in univariate analyses of the derivation cohort with no missing data, including smoking history, UIP/probable UIP, severity of respiratory failure, CT pattern on AE-CFIP, and their combinations. Smoking status and the combination of severe respiratory failure and diffuse pattern were the predictive factors of 90-day mortality (HR 0.36 [IQR 0.14–0.95], p = 0.039 and HR 3.51 [IQR 1.26–9.80], p = 0.016, respectively). In the validation cohort, a combination of severe respiratory failure and diffuse pattern was also predictive for 90-day mortality (HR 4.30 [IQR 1.51–12.2], p = 0.006).

### Sensitivity analysis in the IPF cohort

**Fig 4:**
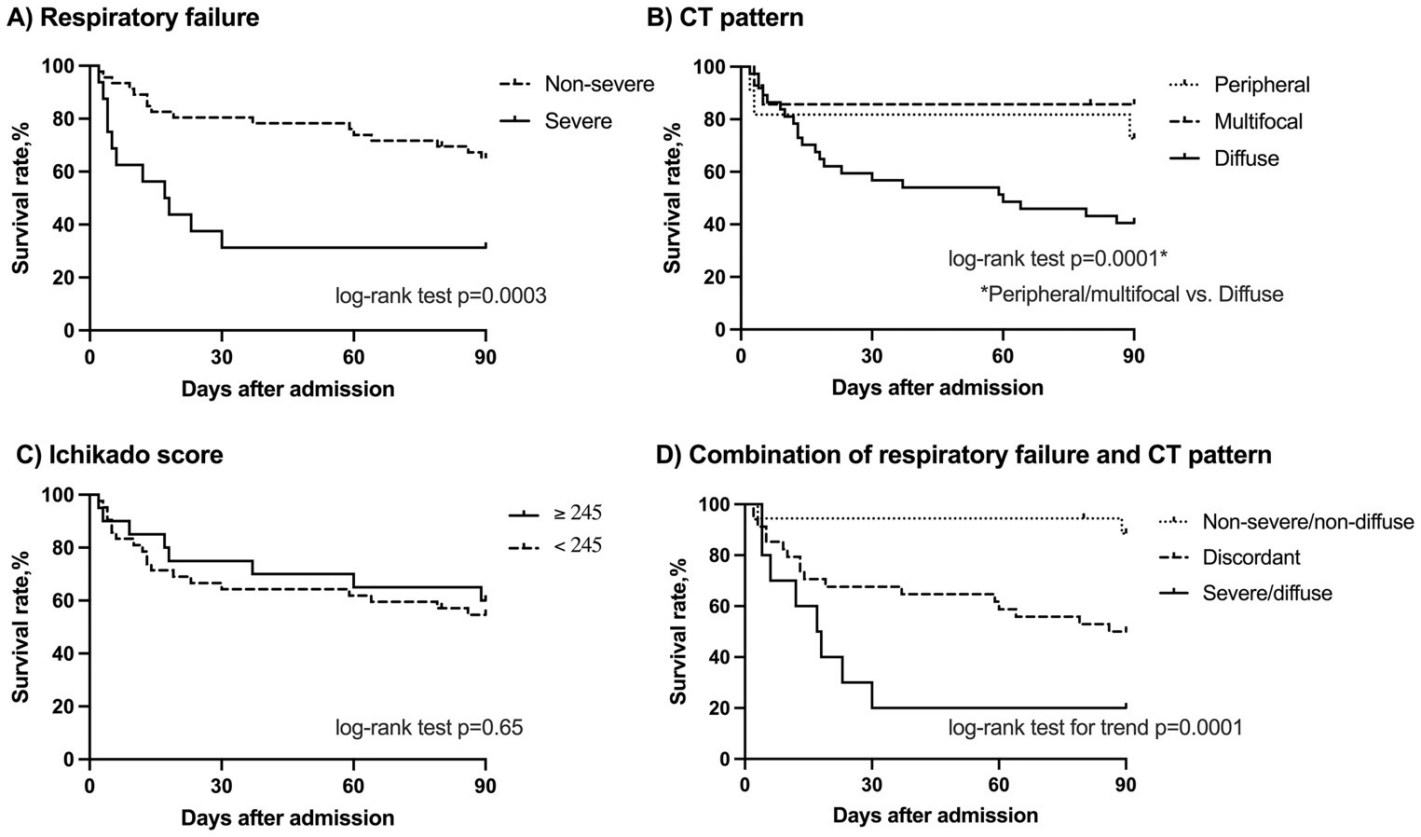
Kaplan-Meier survival curves for patients with acute exacerbation of idiopathic pulmonary fibrosis in the combined dataset of derivation and validation cohorts. (A) Comparison between the patients with non-severe (dotted line, n = 46) and severe (solid line, n = 16) respiratory failure on hospitalization. (B) Comparison by distribution pattern of newly-appeared opacities on computed tomography (CT); peripheral pattern (dashed line, n = 11), multifocal pattern (dotted line, n = 14), and diffuse pattern (solid line, n = 37). (C) Comparison between the patients with Ichikado score ≥ 245 (solid line, n = 20) and < 245 (dotted line, n = 42). (D) Comparison by the combination of severity of respiratory failure and CT pattern in the derivation cohort; non-severe respiratory failure and non-diffuse pattern (dotted line, n = 18), severe respiratory failure and diffuse pattern (solid line, n = 10), and discordant group (dashed line, n = 34).

Sixty-two cases of IPF were identified in the combined dataset of the derivation and validation cohorts. Twenty-eight patients (45%) died within 90 days of admission due to AE-IPF. The respiratory failure grade, CT pattern, and combination of two were predictive of survival within 90 days (Fig 4), as was observed in cases of AE-CFIP.

## Discussion

In our retrospective analysis of 54 cases with AE-CFIP, the severity of respiratory failure defined by FiO_2_ required to maintain SpO_2_ ≥ 90% at hospitalization and diffuse distribution of newly developed ground-glass opacity or consolidation on thoracic HRCT images was significantly associated with 90-day mortality. This was especially noted when the two were analyzed in combination. However, post-AE Ichicado scores that reflect the extent of fibroproliferative changes were not associated with the outcome of AE-CFIP. These findings were confirmed by an additional analysis of 31 cases in the validation cohort. This suggests that the intensity and extent of lung injury during AE at the site where gas exchange can be maintained without structural remodeling is essential for the prognosis of AE-CFIP.

Two methods have been used to evaluate CT patterns in the lungs during AE-IPF; one which focuses on the distribution pattern of newly developed opacities, and the other that evaluates the extent of fibroproliferative changes in greater detail. Our data confirmed the findings of a previous report which documented that diffuse patterns of new lung opacities are associated with poorer prognosis as compared with peripheral or multifocal patterns [8]. The Ichikado score evaluates the extent of ground-glass opacity, consolidation, bronchiectasis, and honeycomb on HRCT images, and has been used to predict the prognosis of patients with ARDS or AE-IPF [9, 10]. However, our study could not find any association between the prognosis of AE-CFIP and the Ichicado score. The discrepancy between the two studies could be possibly attributed, in part, to the extent of fibrotic changes prior to AE. The pre-AE honeycomb score in the cases of previous study was 5.0 [10], whereas the patients in the present study demonstrated more advanced lung fibrosis with a honeycomb score of 30. These results suggest that newly developed lesions that compromise gas exchange, and not the preexisting fibrotic lesions, are more important for the prognosis of AE of advanced CFIP.

The severity of respiratory failure on admission was not significantly associated with the distribution of newly developed ground-glass opacity or consolidation on thoracic CT images; a diffuse pattern being observed in 49% and 53% of cases with non-severe and severe respiratory failure, respectively, in the derivation cohort (p = 0.99). A substantial proportion of patients (28/58 cases, 48%) demonstrated discordance between the severity of respiratory failure and the extent of lung opacities. The prognosis of patients with discordant features was better than those with concordant severe respiratory failure and diffuse CT pattern, suggesting that the severity of respiratory failure was determined not only by the area of the damaged lung, but also by the pathological changes in the injured lung. This hypothesis is supported by observations in cases of ARDS as reported earlier. A post-mortem analysis of 356 cases of ARDS had demonstrated that 56%, 40%, and 12% of the cases with severe, moderate, and mild respiratory failure presented with diffuse alveolar damage, whereas others suffered from organizing pneumonia [29]. Although some studies have shown an association between AE-CFIP prognosis and the degree of hypoxemia and/or the extent of new shadows [2, 18], this is the first study demonstrating that the combination of severe respiratory failure and diffuse pattern is a more informative approach for predicting the short-term prognosis of patients with AE-CFIP.

There are a few limitations to the present study. First, it was a retrospective study conducted in two clinical centers with a small sample size, especially in the validation cohort. However, the obtained results are reliable as data from the first cohort were confirmed in an independent cohort. Second, this study examined a population with mixed backgrounds, including cases of IPF and non-IPF. However, severe respiratory failure and diffuse patterns on CT was associated with a poor prognosis, independent of the presence of UIP/probable UIP. Therefore, in future studies, cases of interstitial pneumonia secondary to collagen vascular diseases and chronic hypersensitivity pneumonia should also be examined.

## Conclusion

Short-term mortality in idiopathic AE-CFIP patients was dependent on the intensity, quality, and area of lung injury, which can be clinically assessed based on the severity of respiratory failure and the distribution of new opacities on HRCT.
